# Insights from Review and Content Analysis of Current COVID-19 Mobile Apps and Recommendations for Future Pandemics

**DOI:** 10.3390/ijerph192214652

**Published:** 2022-11-08

**Authors:** Yeongju Kim, Jihye Choi, Young-A Ji, Hyekyung Woo

**Affiliations:** 1 Department of Health Administration, Kongju National University, Gongju 32588, Korea; 2 Department of Health Promotion and Behavioral Sciences, School of Public Health, University of Texas Health Science Center at Houston, Houston, TX 77030, USA; 3 College of Medicine, Gyeongsang National University, Jinju 52828, Korea; 4 Institute of Health and Environment, Kongju National University, Gongju 32588, Korea

**Keywords:** COVID-19, corona, contact tracing, outbreak detection, coronavirus

## Abstract

A number of mobile health apps related to coronavirus infectious disease 2019 (COVID-19) have been developed, but research into app content analytics for effective surveillance and management is still in its preliminary stages. The present study aimed to identify the purpose and functions of the currently available COVID-19 apps using content analysis. The secondary aim was to propose directions for the future development of apps that aid infectious disease surveillance and control with a focus on enhancing the app content and quality. Prior to conducting an app search in the App Store and the Google Play Store, we reviewed previous studies on COVID-19 apps found in Google Scholar and PubMed to examine the main purposes of the apps. Using the five selected keywords based on the review, we searched the two app stores to retrieve eligible COVID-19 apps including those already addressed in the reviewed literature. We conducted descriptive and content analyses of the selected apps. We classified the purpose types of the COVID-19 apps into the following five categories: Information provision, tracking, monitoring, mental health management, and engagement. We identified 890 apps from the review articles and the app stores: 47 apps met the selection criteria and were included in the content analysis. Among the selected apps, iOS apps outnumbered Android apps, 27 apps were government-developed, and most of the apps were created in the United States. The most common function for the iOS apps (63.6%) and Android apps (62.5%) was to provide COVID-19-related knowledge. The most common function among the tracking apps was to notify users of contact with infected people by the iOS apps (40.9%) and Android apps (37.5%). About 29.5% of the iOS apps and 25.0% of the Android apps were used to record symptoms and self-diagnose. Significantly fewer apps targeted mental health management and engagement. Six iOS apps (6/44, 13.6%) and four Android apps (4/24, 16.7%) provided behavioral guidelines about the pandemic. Two iOS apps (2/44, 4.5%) and two Android apps (2/24, 8.3%) featured communication functions. The present content analysis revealed that most of the apps provided unilateral information and contact tracing or location tracking. Several apps malfunctioned. Future research and development of COVID-19 apps or apps for other emerging infectious diseases should address the quality and functional improvements, which should begin with continuous monitoring and actions to mitigate any technical errors.

## 1. Introduction

The outbreak of coronavirus infectious disease 2019 (COVID-19) has marked an unprecedented global public health crisis with devastating consequences. The public and private health care sectors have made substantial efforts to mitigate the impact of the pandemic including the development of digital surveillance systems to monitor and contact-trace confirmed cases [1,2]. Public health authorities are using technologies to strengthen conventional epidemiological methods [3,4], as recommended by the World Health Organization (WHO) in a recent publication on the use of digital technologies to bolster global health security [5]. An integral part of digital health is the use of mobile wireless technologies, or mobile health (mHealth) [1]. The high penetration rates of smartphones and the concomitant use of mobile applications (hereinafter referred to as apps) have allowed for the real-time collection [2] and sharing of health information, which are the core competencies of apps for pandemic management [6]. Common functions of COVID-19 apps include providing the current status of the pandemic, contact tracing, sending notifications, reporting symptoms following self-diagnosis, and relaying vaccine-related information [7]. COVID-19 apps have demonstrated efficient disease control and are actively used in many countries such as the United States, Japan, and Singapore [7,8]. Korea is no exception, considering the nation’s high smartphone penetration rate. The Korea Disease Control and Prevention Agency successfully launched the COOV app where individuals use this app to obtain their vaccination certification [9].

Despite the advent of new mobile technologies and the burgeoning of COVID-19 apps, several precautions should be addressed for the more effective control of the disease. First, it is necessary to revise existing guidelines for app developers based on the observed weaknesses of the current COVID-19 apps. Strong emphasis should be given to enhancing the reliability of information, protecting personal information, heightening remote surveillance, and minimizing malfunctions. The contents of COVID-19 apps should be rigorously analyzed after improving the guidelines for app development [1,7]. For example, one study reviewed apps based on technical features such as app capacity and data sharing availability to inform on the quality improvements in the apps [8]. Another study examined current COVID-19 apps in light of different target users (quarantined people and travelers), in addition to key functions such as health monitoring, raising awareness, and vaccination management [10]. Research of this kind has been conducted mainly in the United States and Europe [7,11,12], but is still in its nascent stage. There is comparatively little research on the content analysis of apps [8,11,13,14]. Further research is warranted to shift from apps of low functional utility [7] to robust and high-quality apps with optimal content and functional utility. Such progress, followed by increased app usage, will enable a rapid response to infectious diseases and accelerate the transition into the post-pandemic era, in which the pandemic will have been effectively controlled.

Numerous apps are available worldwide to prepare for similar future outbreaks such as apps utilized for telemedicine, symptom monitoring, and alerts to prevent the spread of infection, but the criteria to create successful app contents remain nebulous [7]. The paucity of systematic reviews and content analyses on pandemic-related apps has resulted in a limited understanding of ways to improve the quality of these apps. Therefore, we formulated the following research questions: (1) What are the characteristics and qualities of the currently commercialized COVID-19 apps? and (2) what are the guidelines and criteria to be met when developing effective apps for managing infectious diseases? To address these questions, we aimed to (1) analyze the contents of the COVID-19 apps available in the App store and Google Play store by the main purposes and functions of the apps and (2) provide recommendations for the development of apps for infectious disease management with a focus on enhancing app content and quality, as described in the content analysis.

## 2. Methods

**Figure 1:**
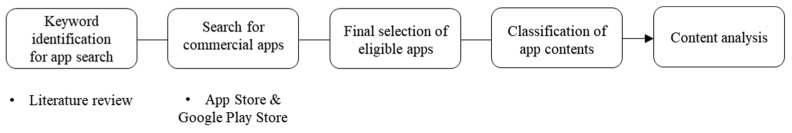
Flowchart of the study.

The process of the study is shown in Figure 1. First, we selected appropriate keywords for the planned app search by reviewing previous review articles related to existing COVID-19 apps. Second, we searched for relevant commercial apps in the App Store and the Google Play Store using the identified keywords from the previous step. Third, we then selected apps that met the eligibility criteria. Fourth, to set the framework for the subsequently content analysis, we explored the review articles and identified the most commonly reported purposes (overall goal of the app; what the app intends to achieve) of the COVID-19 apps across the studies. Fifth, we classified the app contents (features that enable the app to achieve its purpose) into the main purposes. Finally, we conducted content analysis on the selected apps from the app store search, reviewed their characteristics, and classified their contents into one of the purposes we had identified from the literature review.

### 2.1. Selection of COVID-19 Apps for Content Analysis

#### 2.1.1. Literature Review of COVID-19 Mobile Apps

We first searched Google Scholar and PubMed to find studies related to COVID-19 mobile apps published between 2020 and 2022. We conducted an initial review of these studies to select appropriate keywords for the planned app search in the App Store and the Google Play Store. Based on our literature review, we confirmed the following five keywords for the app search: “COVID-19”, “corona”, “contact tracing”, “outbreak detection“, and “coronavirus” [7,12,15,16,17].

#### 2.1.2. Selection of COVID-19 Apps

We searched for apps in the Google Play Store and App store using the five selected keywords including the COVID-19 apps identified in previous studies [1,2,7,8,11,13,14]. We established the inclusion criteria to select apps appropriately based on previous mobile app content analyses [18,19]. To be included in the present study, apps had to be (1) related to COVID-19; (2) free; (3) in Korean or English language; (4) unique without duplicates in the same platform; and (5) operative at the time of the search. Apps that did not meet any one of these criteria were excluded from the study. The raw data in its can be found in Supplementary Material File S1.

### 2.2. Content Analysis of COVID-19 Apps

We referred back to the review articles to explore and identify commonly reported purposes of development and the contents of the existing COVID-19 apps across the studies. After screening and obtaining relevant apps from the app stores and these review articles, we classified the selected apps by various app characteristics such as operating system and developer, and created an app list. We checked whether the main contents classified based on previous research reflected the contents of the final research target apps through content-specific coding. Frequency analysis was conducted to describe the current status of the main purpose and the detailed contents of COVID-19-related apps, which was visualized in Word Cloud.

## 3. Results

### 3.1. Selection of COVID-19 Apps for Content Analysis

**Figure 2:**
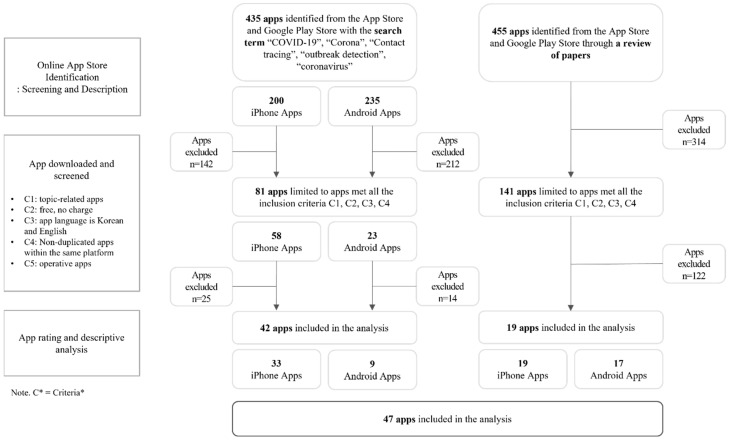
Flowchart of COVID-19 mobile identification; screening and review of COVID-19 mobile apps.

We obtained 890 apps from the App Store, Google Play, and the literature search using the keywords. After excluding apps that did not meet the eligibility criteria, 47 apps were selected as the final study subjects. Figure 2 illustrates the selection process for the apps eligible for full review.

### 3.2. Content Analysis of COVID-19 Apps

We selected the five most cited reviews of COVID-19 apps in the literature to find commonly reported purposes of the apps. Our rationale was that a highly referenced review indicates that it likely reports accurate, reliable, and noteworthy findings as well as holistic trends pertaining to a research area and that many scholars and experts have consulted the review to make further progress in their field of study. We reviewed the COVID-19 apps across the review articles and categorized the contents of the COVID-19 apps into five major types of purpose: “information provision”, “tracking”, “monitoring”, “mental health management”, and “engagement”. Table A1 in Appendix A shows the contents of the apps explored in the previous studies that we categorized by purpose. The tracking purpose was mentioned in all five articles, whereas engagement was mentioned in only two articles (Table A1).

Under each of the five purposes, the specific contents of the COVID-19 apps are listed along with a short description of each content (Table 1). Apps that provided information included health information, live statistics, and the latest news about the COVID-19 pandemic. Apps with a tracking purpose manifested location-sensitive features such as location-specific monitoring of a disease outbreak and alerts to notify when the user was near infected people. Apps that were designed for a monitoring purpose allowed users to record their symptoms and self-diagnose, while apps targeting mental health management provided users with behavioral guidelines during the pandemic. Apps that were designed for the purpose of engagement included financial incentives at mobile app stores and communication channels on social network services.

The basic characteristics of the selected apps such as the operating system, developer, and country of release are summarized in Table 2. A total of 44 iOS apps and 24 Android apps were selected, and 21 apps were available in both operating systems. We classified the apps by provider including government, international organization, public institution, university, and corporate. Among the 47 apps, 27 apps were government-developed, and the greatest number of apps (13/47) were developed in the United States.

**Figure 3:**
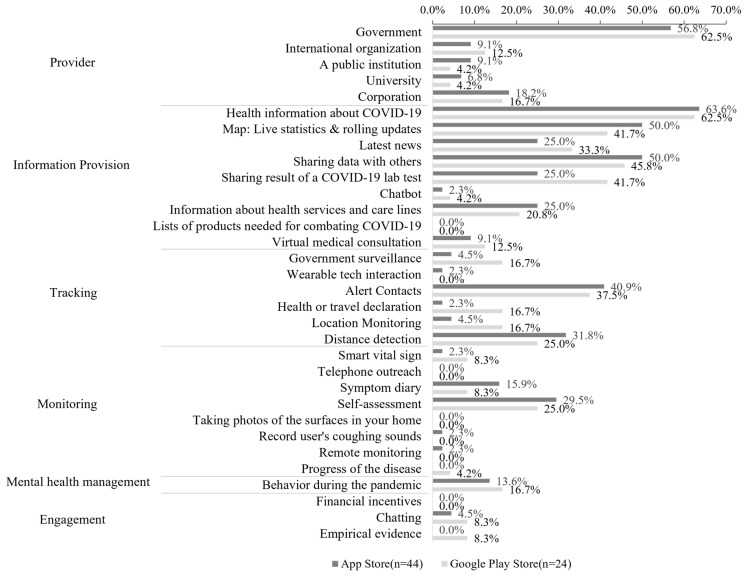
Result of the COVID-19 app content analysis.

**Figure A1:**
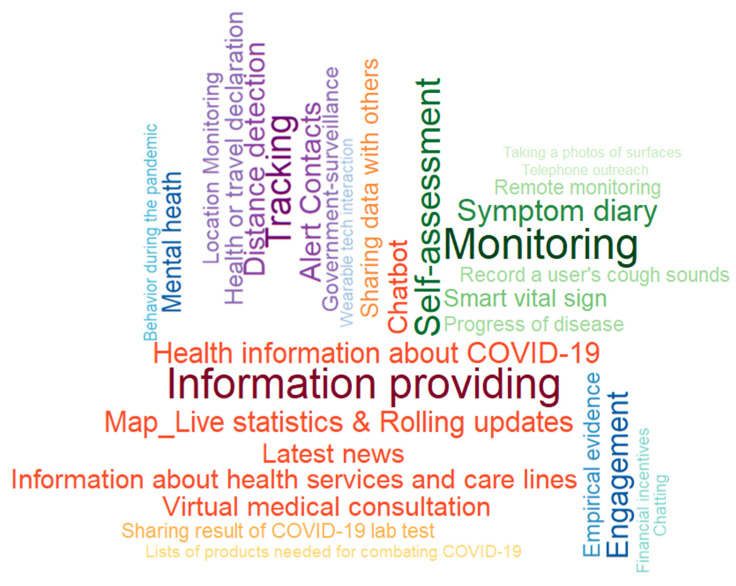
Word Cloud for content frequency.

Figure 3 shows the results of the content analysis for the selected apps. We present a direct comparison of the proportion of apps from the App Store and the Google Play Store by primary purpose and content, which were identified from the review of the review articles on COVID-19 apps. Among the apps with the primary purpose of information provision, the most common app function was to provide COVID-19-related knowledge (iOS apps, 28/44, 63.6% and Android apps, 15/24, 62.5%). Among the tracking apps, the most common app function was to notify users of their contact with infected people (iOS apps, 18/44, 40.9% and Android apps, 9/24, 37.5%). The most common function for the apps with a monitoring purpose was to record symptoms and self-diagnose (iOS apps, 13/44, 29.5% and Android apps, 6/24, 25.0%). Significantly fewer apps were used for mental health management and engagement. Only six iOS apps (6/44, 13.6%) and four Android apps (4/24, 16.7%) provided behavioral guidelines about the pandemic. Two iOS apps (2/44, 4.5%) and two Android apps (2/24, 8.3%) had communication functions available. Figure A1 in Appendix A describes the Word Cloud visualization according to the content analysis.

## 4. Discussion

### 4.1. Principal Findings

Mobile apps for COVID-19 management were used as tools for risk communication during the pandemic, but several were incomplete and malfunctioned [20], indicating that these apps are still in their early stages of development. For example, many of the COVID-19 apps addressed in previous review studies could not be retrieved from the app store because they have since been revoked or withdrawn. To ensure sustained use of the apps for risk communication in infectious disease management, it is necessary to revisit and improve their quality and function, which will involve continuous monitoring and amending the technical errors displayed in these apps. In the present study, we collected and conducted the content analysis of commercially available mobile apps for COVID-19 to examine their purpose, functions, and characteristics and to gain insights into the directions for the development of apps for infectious disease control. Below, we highlight the key findings of our content analysis.

The majority of the selected apps were developed by government agencies and were launched during the initial months of the pandemic in 2020, mainly in developed countries [7,8]. Based on a review of the most cited review articles on the current COVID-19 apps, we identified five major types of purpose including “information provision”, “tracking”, “monitoring”, “mental health management”, and “engagement”. The majority of the apps focused on tracking the infected contacts and providing information to users as a one-sided communication rather than being interactive. The most common functions of apps with the primary purpose of providing information were to provide knowledge related to COVID-19 and generate real-time indicators of COVID-19 activity levels such as statistics on the cases, deaths, test positivity, and hospitalizations. The functions of recently developed COVID-19 apps have expanded from providing only information compatible with social media platforms to supporting contact tracing, so cases can be identified, documented, and quarantined promptly [7]. Nevertheless, the spread of erroneous information remains a concern given the nature of social media platforms, which may lead to the unintended disclosure of personal information during data collection [12]. Several apps support chat functions to facilitate communication, but those that motivated consumers to engage with the app were significantly lacking in quantity. In addition, while previous studies [7,15,21] have emphasized the importance of apps as adjunctive resources for mental health concerns to overcome post-COVID depression, our findings reveal that this content was not prevalent in the apps.

Studies have presented mixed findings about the number and frequency of technical issues reported on the App Store and Google Play; one study reported that more apps from the App Store had problems than those from the Google Play Store [8] whereas the opposite was reported in another study [11]. Numerous irrelevant and malfunctioning apps were seen in the Google Play Store, accounting for 79.1% and 60.9% of the total number of retrieved apps, respectively. We confirmed that there were more unverified Android apps in the Google Play Store compared to iOS apps in the App Store and that some of the Android apps were technically highly unstable such as malfunctions, consistent with the findings of Ming et al. (2020). Thus, more Android apps were excluded from the analysis due to their ineligibility compared to iOS apps. The retrieval of unverified apps was ascribed to the fact that the Google Play Store publishes apps without any quality screening, whereas the App Store reviews guidelines and sets a strict screening for app quality and functionality [22,23]. Thus, the absence of a strict quality screening in the Google Play Store diminishes the average performance of the apps [23].

There is a paucity of empirical evidence on the effectiveness of mobile apps for infectious disease surveillance and control. One way to demonstrate the effectiveness of these mobile apps is to evaluate the app content meticulously and its variability including dubious or unexplored content that may pose quality concerns [24]. The Mobile Application Rating Scale (MARS) and the user Mobile Application Rating Scale (uMARS) are reliable multidimensional tools for assessing the quality of mobile apps that promote health and well-being [25]. Health professionals have utilized these tools to assess apps and to manage risk factors for chronic diseases (such as apps related to diet, physical activity, and stopping smoking). However, among the studies that have used the MARS, fewer studies have assessed apps for COVID-10 or other infectious diseases, compared to those that have assessed apps for chronic disease management [13,26]. Given that the key contents of COVID-19 apps are primarily real-time surveillance and prompt response that may differ from functions of apps for chronic disease management, evaluation tools specifically tailored to apps for infectious diseases management may be more beneficial and practical. It is also important to establish and update clear standards on how to optimally evaluate the quality of these apps for a particular health-related use as they continue to evolve [27]. As mentioned, in a situation such as a pandemic, infectious disease-related apps should provide users with appropriate and prompt content. Since the information can have a critical effect on app users, it is considered to be very important to manage the quality of the content. The quality of the contents includes issues such as prompt and reliable information provision, communication, motivation that can induce app participation, mental health, and other issues. In the case of apps used in a pandemic situation, they play a very important role such as being used to quickly check the traces of infected people and close contacts, prevent the spread of the virus, and quickly implement public health response. Therefore, it can be seen that the management of the quality of the content has a very important meaning. To combat this potential drawback, it is necessary to implement plans and develop evaluation scales tailored to the app content related to infectious disease surveillance and outbreak management.

In addition, government-created or supported apps for risk communication are anticipated to be in greater demand if they can be publicized with easier access and use. The U.S. Centers for Disease Control and Prevention (CDC) recently launched 29 risk communication apps, of which 11 are for general users and nine are for health care providers [28]. “Solve the Outbreak” is a popular user-centered game-based app designed to provide information on infectious diseases and has received the highest ratings and review among the apps launched by the CDC. Previous research has reported that the emerging gamification strategy has a potential impact on health awareness campaigns to fight COVID-19 [27]. Information such as the threat posed by infectious diseases, the manner in which they are spread, and various preventive measures may be challenging to communicate during the surge of an epidemic or pandemic [29]. Game-based apps can deliver important COVID-19 information and messages in a way that more readily resonates with users, ultimately motivating the public to proactively engage in preventive behaviors to defeat the pandemic at its early onset [27]. While this app is exemplary in demonstrating the importance of developing apps available for the general population, its areas for improvement also indicate that further research on app content is needed to promote continued app use.

### 4.2. Recommendations for COVID-19 Apps

The results of the COVID-19 app content analysis will serve as a valuable reference for growth in mobile risk communication apps related to emerging infectious diseases. Based on our findings, we offer the following recommendations for the future development of mobile apps for infectious disease surveillance and outbreak management (Box 1).

Box 1 **Recommendations for COVID-19 apps.**

1. From the initial stage of app development, continuous monitoring should be facilitated to address technical problems and to take immediate action to supplement the maintenance of the apps.

2. App developers should leverage motivational and entertaining elements such as content sharing via social network services and gaming features for increased user engagement, sustained use of the apps, and behavior change to defeat the pandemic.

3. App developers should consistently devise plans to modify and re-engineer the apps for quality improvement of content related to strategic risk communication for infectious disease outbreaks.

4. App developers should build a virtual walk-through for the apps along with the necessary information to maximize their perceived ease of use.

### 4.3. Limitations

This study had several limitations. First, the search for COVID-19 apps analyzed in this study was conducted for a limited time. Given the irregular updates in the app store market and frequent changes in the app contents, the contents at the time of conducting this analysis may have differed slightly from the currently available contents. Second, no quality assessment was considered in the literature review or the content analysis, which focused on the purpose, functions, and characteristics of the selected apps. Future researchers may want to conduct quality assessments on mobile apps for infectious disease surveillance and outbreak surveillance using expert and user-centered evaluation scales. We also suggest that future research compare the perspectives of experts and users on the extent to which they are satisfied with the quality of the app content.

## 5. Conclusions

This study aimed to identify the main purpose, functions, and characteristics of current COVID-19 apps and to provide recommendations for the future development of apps related to infectious disease surveillance and control. We conducted a content analysis of commercially available COVID-19 apps in the App Store and Google Play Store. Our analysis revealed that most of the apps provided unilateral information and contact tracing or location tracking; several apps malfunctioned. Future research and development of COVID-19 apps or apps for other emerging infectious diseases should address the app quality and improve the functions, which begins with continuous monitoring and timely actions to mitigate any technical errors.

## Figures and Tables

**Table 1:** Overview of the COVID-19 apps from the literature review.

Purpose	Content	Description
Information provision	Health information about COVID-19	Provide health guideline information related to COVID-19
	Map: Live statistics and rolling updates	Statistics by region that can be checked with map information (GIS etc.,) and real-time number of confirmed cases
	Latest news	The latest news about COVID-19 and health
	Sharing data with others	Capability of building a health diary describing the users’ symptoms and allowing consumers to share their COVID-19 stories with other users
	Sharing result of a COVID-19 lab test	Share the results of COVID-19-related research
	Chatbot	Chatbots are artificial intelligence (AI)-enabled agents that connect with patients through texting or a human-like voice
	Information about health services and care lines	Helplines connect users to consultants who provide useful information related to COVID-19 and facilitate the introduction of patients to healthcare workers on a toll-free number
	Lists of products needed for combating COVID-19	Lists of products required for combating COVID-19 (e.g., gowns, surgical masks, respirators, face shields, and hand sanitizer)
	Virtual medical consultation	Virtual medical consultation, live video consultations, or bidirectional text-audio communications to provide personalized support between users and their doctors
Tracking	Government surveillance	e.g., tracking through automated teller machine transactions and surveillance data
	Wearable tech interaction	Communication of daily mood and health status through AI voice on a wearable device such as a smartwatch
	Alert Contacts	Notification when contacting infected people based on technology, such as Bluetooth and GPS
	Health or travel declaration	1. Report yourself and your family and record your daily health status for 14 days after arriving at your travel destination. 2. Some of this content notified users of potential exposure risks in the area where they lived
	Location Monitoring	The user selects a specific area from the local options and checks the infectious disease situation in that area in real-time
	Distance detection	Improves the user’s ability to avoid close contact with other people around them
Monitoring	Smart vital sign	Check your heart rate, breathing, etc.
	Telephone outreach	Remote monitoring of self-isolated patients using telephone outreach
	Symptom diary	Records and checks your symptoms daily
	Self-assessment	Evaluates your physical condition using a self-questionnaire
	Taking photos of the surfaces in your home	Inspecting the surface by photography to track hygiene practices
	Record user’s coughing sounds	Recording the coughing sounds of the user
	Remote monitoring	Health care professionals monitor the patients’ health data such as heart rate and blood oxygen level in real-time
	Progress of the disease	Develop a predictive model about disease progression as well as track disease progress in real-time
Mental health management	Behavior during the pandemic	Provide behavioral guidelines and information for mental healthcare during the COVID-19 pandemic
Engagement	Financial incentives	e.g., offers of free credit at the mobile app store.
	Chatting	e.g., connecting to SNS, communicating with users.
	Empirical evidence	Real life estimated treatment effects, precise descriptions of the context in which the tool was used.

**Table 2:** List of apps for final analysis.

No	App Name	OS		Provider					Country	Confirmation Path	
		iOS	Android	Government	International Organizations	A Public Institution	University	Corporation		Literature	App Store
1	안전디딤돌 ^a^	✔		✔					Korea		✔
2	COVID-19 Sounds	✔					✔		United Kingdom		✔
3	코로나19 지침 검색 ^b^	✔	✔			✔			Korea		✔
4	HEALTHLYNKED COVID-19 Tracker	✔						✔	United States		✔
5	CDC	✔	✔		✔				United States	✔	✔
6	COVID-19 UAE	✔		✔					United Arab Emirates	✔	✔
7	Coronavirus Australia	✔		✔					Australia		✔
8	Tarassud+	✔		✔					Oman		✔
9	COVID-19: Happy & Healthy	✔						✔	United Kingdom		✔
10	COVID-19: Response	✔			✔				UN		✔
11	Tabaud (COVID-19 KSA)	✔	✔	✔					Saudi Arabia	✔	✔
12	Coronavirus—COVID-19	✔						✔	Russia		✔
13	HowWeFeel	✔	✔					✔	United States	✔	✔
14	COVID-19 and flu information	✔		✔					United Kingdom		✔
15	BC COVID-19 Support	✔				✔			Canada		✔
16	WHO Academy: COVID-19 Learning	✔			✔				WHO		✔
17	Infected—COVID-19 NL	✔						✔	Netherlands		✔
18	검역신고 · 자가진단—질병관리청 ^c^	✔	✔	✔					Korea	✔	✔
19	MyAus COVID-19	✔				✔			Australia		✔
20	인천광역시교육청 코로나19 꼼짝 마! ^d^	✔				✔			Korea		✔
21	JamCOVID-19	✔	✔	✔					Jamaica	✔	✔
22	코로나 동선 안심이 ^e^	✔	✔					✔	Korea		✔
23	COVID Alert South Africa	✔		✔					Republic of South Africa		✔
24	COVID Alert NY	✔		✔					United States		✔
25	COVID Alert NJ	✔		✔					United States		✔
26	Guam COVID Alert	✔		✔					Guam		✔
27	COVID Alert Malta	✔		✔					Malta		✔
28	COVID Watch Arizona	✔	✔	✔					United States	✔	✔
29	SlowCOVIDNC	✔		✔					United States		✔
30	Jersey COVID Alert	✔	✔	✔					United Kingdom		✔
31	COVID Defense	✔		✔					United States		✔
32	CoCare App	✔	✔	✔					Pakistan	✔	✔
33	TRACE COVID-19	✔					✔		United States		✔
34	Rakning C-19	✔	✔	✔					Iceland	✔	
35	NOVID	✔	✔					✔	United States	✔	
36	CovTracer—EN	✔	✔	✔					Cyprus	✔	
37	Immuni	✔	✔	✔					Italy	✔	
38	COVIDWISE	✔	✔	✔					United States	✔	
39	GuideSafe	✔	✔	✔					United States	✔	
40	OpenWHO	✔	✔		✔				Germany, WHO	✔	
41	WebMD: Symptoms, Rx, & Doctors	✔	✔					✔	United States	✔	
42	MyGov India	✔	✔	✔					India	✔	
43	CoronaReport	✔	✔				✔		United Kingdom	✔	
44	COVID Tracker Ireland	✔	✔	✔					Ireland	✔	✔
45	WHO Info		✔		✔				WHO		✔
46	SMC COVID-19 Tracker		✔	✔					India		✔
47	COVID-19-DXB smart app		✔	✔					Dubai		✔

Korean is the name of a APP. An app can be simply described as: ^a^ Providing various disaster safety information; ^b^ Search and provide COVID-19 guidance; ^c^ Quarantine report and self-diagnosis; ^d^ COVID-19 information provision; ^e^ Confirmation of contact with an infected person.

## Data Availability

Not applicable.

## Supplementary Materials

The following supporting information can be downloaded at: https://www.mdpi.com/article/10.3390/ijerph192214652/s1, File S1: The raw data used in the study.

## Appendix A

**Table A1:** Commonly reported purposes of the COVID-19 apps identified in the most cited relevant reviews.

	Information Provision	Tracking	Monitoring	Mental Health Management	Engagement
Manal Almalki, 2021 [7] ^a^	✔	✔	✔	✔	
Hanson John Leon Singh, 2020 [12] ^b^	✔	✔	✔		
Haridimos Kondylakis, 2020 [15] ^c^	✔	✔	✔	✔	
Esli Osmanlliu, 2021 [16] ^d^		✔			✔
Nouf Sahal Alharbi, 2022 [17] ^e^	✔	✔	✔	✔	✔

^a^ Health Apps for Combating COVID-19: Descriptive Review and Taxonomy; ^b^ Mobile Health Apps That Help With COVID-19 Management: Scoping Review; ^c^ COVID-19 Mobile Apps: A Systematic Review of the Literature; ^d^ Considerations for the Design and Implementation of COVID-19 Contact Tracing Apps: Scoping Review; ^e^ COVID-19 Mobile Apps in Saudi Arabia: Systematic Identification, Evaluation, and Features Assessment.

## References

[1] Collado-Borrell R., Escudero-Vilaplana V., Villanueva-Bueno C., Herranz-Alonso A., Sanjurjo-Saez M. (2020). Features and Functionalities of Smartphone Apps Related to COVID-19: Systematic Search in App Stores and Content Analysis. J. Med. Internet Res..

[2] Wirth F.N., Johns M., Meurers T., Prasser F. (2020). Citizen-Centered Mobile Health Apps Collecting Individual-Level Spatial Data for Infectious Disease Management: Scoping Review. JMIR Mhealth Uhealth.

[3] Ukpong I., Etim S., Ana P. (2020). Application of Mobile Technology in Community Transmission Surveillance for 2020 Corona Virus Disease.

[4] Alwashmi M.F. (2020). The Use of Digital Health in the Detection and Management of COVID-19. Int. J. Environ. Res. Public Health.

[5] Recommendations on Digital Interventions for Health System Strengthening. https://www.who.int/publications/i/item/9789241550505.

[6] Aslani N., Lazem M., Mahdavi S., Garavand A. (2020). A Review of Mobile Health Applications in Epidemic and Pandemic Outbreaks: Lessons Learned for COVID-19. Arch. Clin. Infect. Dis..

[7] Almalki M., Giannicchi A. (2021). Health Apps for Combating COVID-19: Descriptive Review and Taxonomy. JMIR Mhealth Uhealth.

[8] Ming L.C., Untong N., Aliudin N.A., Osili N., Kifli N., Tan C.S., Goh K.W., Ng P.W., Al-Worafi Y.M., Lee K.S. (2020). Mobile Health Apps on COVID-19 Launched in the Early Days of the Pandemic: Content Analysis and Review. JMIR Mhealth Uhealth.

[9] About COOV. https://ncv.kdca.go.kr/menu.es?mid=a12501000000.

[10] Lee B., Ibrahim S.A., Zhang T. (2021). Mobile Apps Leveraged in the COVID-19 Pandemic in East and South-East Asia: Review and Content Analysis. JMIR Mhealth Uhealth.

[11] Elkhodr M., Mubin O., Iftikhar Z., Masood M., Alsinglawi B., Shahid S., Alnajjar F. (2021). Technology, Privacy, and User Opinions of COVID-19 Mobile Apps for Contact Tracing: Systematic Search and Content Analysis. J. Med. Internet Res..

[12] John Leon Singh H., Couch D., Yap K. (2020). Mobile Health Apps that Help with COVID-19 Management: Scoping Review. JMIR Nurs..

[13] Davalbhakta S., Advani S., Kumar S., Agarwal V., Bhoyar S., Fedirko E., Misra D.P., Goel A., Gupta L., Agarwal V. (2020). A Systematic Review of Smartphone Applications Available for Corona Virus Disease 2019 (COVID19) and the Assessment of their Quality Using the Mobile Application Rating Scale (MARS). J. Med. Syst..

[14] Mohanty B., Chughtai A., Rabhi F. (2019). Use of Mobile Apps for epidemic surveillance and response—Availability and gaps. Glob. Biosecurity.

[15] Kondylakis H., Katehakis D.G., Kouroubali A., Logothetidis F., Triantafyllidis A., Kalamaras I., Votis K., Tzovaras D. (2020). COVID-19 Mobile Apps: A Systematic Review of the Literature. J. Med. Internet Res..

[16] Osmanlliu E., Rafie E., Bédard S., Paquette J., Gore G., Pomey M.-P. (2021). Considerations for the Design and Implementation of COVID-19 Contact Tracing Apps: Scoping Review. JMIR mHealth uHealth.

[17] Alharbi N.S., Alsubki N., Altamimi S.R., Alonazi W., Fahlevi M. (2022). COVID-19 Mobile Apps in Saudi Arabia: Systematic Identification, Evaluation, and Features Assessment. Front. Public Health.

[18] Lee J.B., Woo H. (2019). Quality evaluation of smartphone applications for physical activity promotion. Korean J. Health Educ. Promot..

[19] Lee J.B., Kim J.H., Bok J.H., Woo H. (2020). Quality Analysis of Smart Application Contents for the Convenience of Care and Hospital Access. Korea J. Hosp. Manag..

[20] Paek H.J. (2017). Strategic risk communication for infectious disease outbreaks: The evolving landscape of publics and media. J. Korean Med. Assoc..

[21] Jaworski B.K., Taylor K., Ramsey K.M., Heinz A., Steinmetz S., Pagano I., Moraja G., Owen J.E. (2021). Exploring Usage of COVID Coach, a Public Mental Health App Designed for the COVID-19 Pandemic: Evaluation of Analytics Data. J. Med. Internet Res..

[22] Meacham M.C., Vogel E.A., Thrul J. (2020). Vaping-Related Mobile Apps Available in the Google Play Store After the Apple Ban: Content Review. J. Med. Internet Res..

[23] Comino S., Manenti F.M., Mariuzzo F. (2019). Updates management in mobile applications: iTunes versus Google Play. J. Econ. Manag. Strategy.

[24] Wyatt J.C. (2018). How can clinicians, specialty societies and others evaluate and improve the quality of apps for patient use?. BMC Med..

[25] Cai X., Zhang F., Lin C., Zhang X., Wang Z., Xing H., Nie L., Han X., Ji L. (2020). Achieving Effective and Efficient Basal Insulin Optimal Management by Using Mobile Health Application (APP) for Type 2 Diabetes Patients in China. Diabetes Metab. Syndr. Obes. Targets Ther..

[26] Salehinejad S., Niakan Kalhori S.R., Hajesmaeel Gohari S., Bahaadinbeigy K., Fatehi F. (2021). A review and content analysis of national apps for COVID-19 management using Mobile Application Rating Scale (MARS). Inform. Health Soc. Care.

[27] Agarwal P., Gordon D., Griffith J., Kithulegoda N., Witteman H.O., Sacha Bhatia R., Kushniruk A.W., Borycki E.M., Lamothe L., Springall E. (2021). Assessing the quality of mobile applications in chronic disease management: A scoping review. NPJ Digit. Med..

[28] Solve the Outbreak App. https://www.cdc.gov/mobile/applications/sto/index.html.

[29] Robinson L.A., Turner I.J., Sweet M.J. (2018). The use of gamification in the teaching of disease epidemics and pandemics. FEMS Microbiol. Lett..

